# Clinical effectiveness and safety of olaparib in BRCA-mutated, HER2-negative metastatic breast cancer in a real-world setting: final analysis of LUCY

**DOI:** 10.1007/s10549-023-07165-x

**Published:** 2023-12-19

**Authors:** Judith Balmaña, Peter A. Fasching, Fergus J. Couch, Suzette Delaloge, Intidhar Labidi-Galy, Joyce O’Shaughnessy, Yeon Hee Park, Andrea F. Eisen, Benoit You, Hughes Bourgeois, Anthony Gonçalves, Zoe Kemp, Angela Swampillai, Tomasz Jankowski, Joo Hyuk Sohn, Elena Poddubskaya, Guzel Mukhametshina, Sercan Aksoy, Constanta V. Timcheva, Tjoung-Won Park-Simon, Antonio Antón-Torres, Ellie John, Katherine Baria, Isabel Gibson, Karen A. Gelmon, The LUCY investigators, Tatyana Koynova, Vasil Popov, Constanta Timcheva, Antoaneta Tomova, Andrea Eisen, Karen Gelmon, Julie Lemieux, Paule Augereau, Fernando Bazan, Célia Becuwe, Hugues Bourgeois, Camille Chakiba, Mohamad Chehimi, Caroline Cheneau, Florence Dalenc, Eléonore de Guillebon, Thibault de La Motte Rouge, Jean-Sébastien Frenel, Anthony Gonçalves, Julien Grenier, Anne Claire Hardy-Bessard, Regine Lamy, Christelle Levy, Alain Lortholary, Audrey Mailliez, Jacques Medioni, Anne Patsouris, Dominique Spaeth, Luis Teixeira, Isabelle Tennevet, Laurence Venat-Bouvet, Cristian Villanueva, Benoit You, Johannes Ettl, Peter Fasching, Bernd Gerber, Claus Alexander Hanusch, Oliver Hoffmann, Tjoung-Won Park-Simon, Wolfram Malter, Mattea Reinisch, Joke Tio, Pauline Wimberger, Katalin Boer, Magdolna Dank, Alberto Ballestrero, Giampaolo Bianchini, Laura Biganzoli, Roberto Bordonaro, Francesco Cognetti, Enrico Cortesi, Michelino De Laurentiis, Sabino De Placido, Luca Gianni, Valentina Guarneri, Paulo Marchetti, Filippo Montemurro, Anna Maria Mosconi, Giuseppe Naso, Fabio Puglisi, Armando Santoro, Claudio Zamagni, Hiroji Iwata, Seung-Jin Kim, Seigo Nakamura, Yee Soo Chae, Eun Kyung Cho, Jee Hyun Kim, Seock-Ah Im, Keun Seok Lee, Yeon Hee Park, Joo Hyuk Sohn, Tomasz Byrski, Tomasz Huzarski, Tomasz Jankowski, Bozena Kukielka-Budny, Aleksandra Lacko, Zbigniew Nowecki, Elzbieta Senkus-Konefka, Renata Szoszkiewicz, Rafal Tarnawski, Timur Andabekov, Mikhail Dvorkin, Viktoria Dvornichenko, Fedor Moiseenko, Guzel Mukhametshina, Elena Poddubskaya, Ekaterina Popova, Anna Tarasova, Dina Sakaeva, Marina Shomova, Anna Vats, Bárbara Adamo, Raquel Andrés Conejero, Antonio Antón Torres, Judith Balmaña Gelpi, Blanca Cantos Sánchez de Ibarguen, Josefina Cruz Jurado, Nieves Díaz Fernández, Alejandro Falcón González, Juan Garcia, Santiago González Santiago, Fernando Henao Carrasco, Isabel Lorenzo Lorenzo, Fernando Moreno Antón, Beatriz Rojas García, Salomón Menjón Beltrán, Marta Santisteban, Agostina Stradella, Ming-Feng Hou, Chiun-Sheng Huang, Yung-Chang Lin, Ling-Ming Tseng, Hwei-Chung Wang, Sercan Aksoy, Cagatay Arslan, Mehmet Artac, Adnan Aydiner, Umut Disel, Metin Ozkan, Ozgur Ozyilkan, Emel Yaman Sezer, Tarkan Yetisyigit, Anne Armstrong, Sophie Barrett, Annabel Borley, Zoe Kemp, Caroline Michie, Mukesh Mukesh, Timothy Perren, Angela Swampillai, Madhu Chaudhry, Tammy Young

**Affiliations:** 1 Medical Oncology Department, Vall d’Hebron University Hospital and Vall d’Hebron Institute of Oncology, Barcelona, Spain; 2 Department of Gynecology and Obstetrics, University Hospital Erlangen, Comprehensive Cancer Center Erlangen-EMN, Friedrich-Alexander University Erlangen-Nuremberg, Erlangen, Germany; 3 Department of Laboratory Medicine and Pathology, Mayo Clinic, Rochester, MN USA; 4 Breast Cancer Unit, Department of Cancer Medicine, Gustave Roussy, Villejuif, France; 5 Department of Oncology, Geneva University Hospital, Department of Medicine, Division of Oncology, Faculty of Medicine, University of Geneva, Geneva, Switzerland; 6 Baylor University Medical Center, Texas Oncology and US Oncology, Dallas, TX USA; 7 Samsung Medical Center, Sungkyunkwan University School of Medicine, Seoul, Republic of Korea; 8 Division of Medical Oncology, Odette Cancer Centre, Sunnybrook Health Sciences Centre, Toronto, ON Canada; 9 Department of Medical Oncology, Hospices Civils of Lyon Cancer Institute, Centre for Therapeutic Investigation in Oncology and Haematology of Lyon, Lyon Sud Hospital Centre, Lyon, France; 10 Faculty of Medicine of Lyon Sud, Claude Bernard Lyon 1 University, Lyon, France; 11 GINECO-GINEGEPS, Paris, France; 12 Medical Oncology Department, Victor Hugo Clinic–Jean Bernard Center, Le Mans, France; 13 Department of Medical Oncology, Institut Paoli-Calmettes, Marseille, France; 14 Cancer Research Center of Marseille, Aix-Marseille University, French National Centre for Scientific Research, National Institute for Health and Medical Research, Marseille, France; 15 Breast Cancer Unit, The Royal Marsden NHS Foundation Trust, London, UK; 16 Department of Clinical Oncology, Guy’s and St Thomas’ Hospital NHS Foundation Trust, London, UK; 17 Breast Cancer Now Research Unit, Guy’s Hospital, King’s College London, London, UK; 18 Department of Pneumology, Oncology and Allergology, Medical University of Lublin, Lublin, Poland; 19 Division of Medical Oncology, Yonsei Cancer Center, Yonsei University College of Medicine, Seoul, Republic of Korea; 20 Surgical Department N2, Clinical Center VitaMed, Moscow, Russia; 21 Ministry of Health of the Republic of Tatarstan, Kazan, Russia; 22 Medical Oncology Department, Hacettepe University Cancer Institute, Ankara, Turkey; 23 Medical Oncology Department, MHAT Nadezhda, Sofia, Bulgaria; 24 Frauenklinik, Hannover Medical School, Hannover, Germany; 25 Department of Medical Oncology, Miguel Servet University Hospital and Aragon Health Research Institute, Zaragoza, Spain; 26 Statistics, AstraZeneca, Cambridge, UK; 27 US Medical Affairs, AstraZeneca, Gaithersburg, MD USA; 28 Global Medical Affairs, AstraZeneca, Cambridge, UK; 29 Department of Medical Oncology, BC Cancer, University of British Columbia, Vancouver, Canada

**Keywords:** Breast cancer 1 gene product, Breast cancer 2 gene product, Breast cancer, Olaparib, Kaplan–Meier survival curves, Progression-free survival, Overall survival

## Abstract

**Purpose:**

The interim analysis of the phase IIIb LUCY trial demonstrated the clinical effectiveness of olaparib in patients with germline BRCA-mutated (gBRCAm), human epidermal growth factor receptor 2 (HER2)-negative metastatic breast cancer (mBC), with median progression-free survival (PFS) of 8.11 months, which was similar to that in the olaparib arm of the phase III OlympiAD trial (7.03 months). This prespecified analysis provides final overall survival (OS) and safety data.

**Methods:**

The open-label, single-arm LUCY trial of olaparib (300 mg, twice daily) enrolled adults with gBRCAm or somatic BRCA-mutated (sBRCAm), HER2-negative mBC. Patients had previously received a taxane or anthracycline for neoadjuvant/adjuvant or metastatic disease and up to two lines of chemotherapy for mBC.

**Results:**

Of 563 patients screened, 256 (gBRCAm, *n* = 253; sBRCAm, *n* = 3) were enrolled. In the gBRCAm cohort, median investigator-assessed PFS (primary endpoint) was 8.18 months and median OS was 24.94 months. Olaparib was clinically effective in all prespecified subgroups: hormone receptor status, previous chemotherapy for mBC, previous platinum-based chemotherapy (including by line of therapy), and previous cyclin-dependent kinase 4/6 inhibitor use. The most frequent treatment-emergent adverse events (TEAEs) were nausea (55.3%) and anemia (39.2%). Few patients (6.3%) discontinued olaparib owing to a TEAE. No deaths associated with AEs occurred during the study treatment or 30-day follow-up.

**Conclusion:**

The LUCY patient population reflects a real-world population in line with the licensed indication of olaparib in mBC. These findings support the clinical effectiveness and safety of olaparib in patients with gBRCAm, HER2-negative mBC.

**Clinical trial registration:**

Clinical trials registration number: NCT03286842

**Supplementary Information:**

The online version contains supplementary material available at 10.1007/s10549-023-07165-x.

## Introduction

Loss-of-function mutations in the breast cancer (BC) susceptibility genes 1 and 2 (*BRCA1* and *BRCA2*; BRCA) are associated with an increased risk of developing BC [1, 2]. Patients with a germline BRCA mutation are often young at initial BC diagnosis and present with aggressive disease [3]. Germline BRCA mutations have been detected in approximately 9.7% of patients with human epidermal growth factor receptor 2 (HER2)-negative metastatic BC (mBC), with prevalence being higher in patients with triple-negative BC (TNBC; 10–20%) than in those with hormone receptor (HR)-positive BC (2–8%) [4–6]. However, with the relative prevalence of the two subtypes, the majority of patients with germline BRCA-mutated (gBRCAm) BC are HR-positive. Due to the hereditary component of gBRCAm BC, the prevalence is higher at approximately 23% in those with a family history of BC or ovarian cancer [5].

The BRCA1 and BRCA2 proteins play critical roles in DNA damage response pathways, particularly in the repair of DNA double-strand breaks [7]. Tumor cells lacking functional BRCA1 or BRCA2 proteins show increased sensitivity to DNA-damaging agents, including poly(ADP-ribose) polymerase (PARP) enzyme inhibitors, as well as platinum-based and non-platinum-based chemotherapies [8–12].

Two phase III randomized clinical studies have delivered robust evidence regarding the efficacy and safety of PARP inhibitors in patients with gBRCAm, HER2-negative locally advanced and/or mBC: OlympiAD (olaparib versus physician’s choice of chemotherapy in mBC, NCT02000622) and EMBRACA (talazoparib versus physician’s choice of chemotherapy in locally advanced and mBC, NCT01945775) [13–19]. Subsequently, both PARP inhibitors were approved as targeted treatments for patients with gBRCAm, HER2-negative mBC (and locally advanced BC in Europe; talazoparib is also approved for locally advanced BC in the USA) who have previously been treated with chemotherapy in the neoadjuvant, adjuvant, or metastatic setting [15, 20–23].

In the OlympiA study (NCT02032823), 1 year of adjuvant olaparib treatment after completion of neoadjuvant or adjuvant chemotherapy and local treatment resulted in significantly longer invasive and distant disease-free survival and fewer deaths compared with placebo in patients with gBRCAm, HER2-negative, high-risk early BC [24, 25]. Based on these findings, olaparib was approved as an adjuvant treatment for patients with gBRCAm, HER2-negative, high-risk early BC who have previously been treated with neoadjuvant or adjuvant chemotherapy [21, 26].

The phase IIIb LUCY trial (NCT03286842) has further demonstrated the clinical effectiveness and well-tolerated safety profile of olaparib in patients with gBRCAm, HER2‑negative mBC, in a setting designed to reflect routine clinical practice more closely than the OlympiAD trial [15, 27]. Encouraging data have suggested that patients with a somatic BRCA mutation (sBRCAm) may benefit from PARP inhibition in the ovarian cancer, prostate cancer, and mBC settings [28–32]. Accordingly, the LUCY trial also permitted enrollment of patients with a sBRCAm [27]. At the data cutoff for the prespecified interim analysis (September 23, 2019), median progression-free survival (PFS; 8.11 months) was consistent with that reported for olaparib in the OlympiAD trial (7.03 months), and no new safety signals were observed [15, 27]. This final planned analysis (data cutoff September 1, 2021) includes assessments of overall survival (OS) and safety.

## Patients and methods

### Study design and treatment

Details of the LUCY trial have been reported previously [27]. In brief, this open-label, single-arm, multicenter, international, phase IIIb study enrolled adults with a gBRCAm or sBRCAm and HER2-negative mBC (triple-negative or HR-positive). Patients with a sBRCAm were permitted following a study protocol amendment (April 27, 2018). Eligible patients had received a maximum of two lines of prior chemotherapy for mBC and either taxane- or anthracycline-based chemotherapy in any setting. Patients treated with prior platinum-based chemotherapy and patients with stable brain metastases were eligible. Those with HR-positive mBC who had previously completed at least one line of endocrine therapy in either an adjuvant or metastatic setting and were considered unsuitable for further endocrine therapy were eligible.

Patients received olaparib tablets (300 mg twice daily) until disease progression, unacceptable toxicity, or other protocol-specified discontinuation criteria were met. Patients who discontinued study treatment were followed to record progression (if treatment was discontinued in the absence of progression), use of subsequent anti-cancer therapies, time to second progression or death (PFS2), and OS.

## Study outcomes and assessments

Tumor assessments were performed at each study visit, up to the first instance of disease progression, and then in accordance with local practice. The primary outcome was investigator-assessed PFS in the gBRCAm cohort, defined as the time from first dose of olaparib to the date of progression or death from any cause. Physician-defined clinical response could be radiologic (as per Response evaluation criteria in solid tumors [RECIST] version 1.1) or symptomatic, or clear progression of non-measurable disease, if progression could be documented.

Secondary clinical effectiveness outcomes (assessed in the gBRCAm cohort) were: OS (time from first dose of olaparib to the date of death from any cause); time to first subsequent treatment or death (TFST; time from first dose of olaparib to first subsequent treatment commencement or death); time to study treatment discontinuation or death (TDT); time to second subsequent treatment or death (TSST); PFS2 (time from first dose of olaparib to the earliest progression event after the event used for the primary endpoint or death from any cause); clinical response rate (CRR); and duration of clinical response (DoCR; time from when the investigator first assessed the patient as responding to the date of progression or death from any cause, in the absence of progression). Time to onset of a clinical response in patients in the gBRCAm cohort was assessed in a *post hoc* analysis. Clinical effectiveness outcomes assessed in the sBRCAm cohort were exploratory.

Tolerability and safety were secondary outcomes. Actual treatment duration was calculated by considering the duration of dose interruptions. Adverse events (AEs) were graded according to National Cancer Institute Common Terminology Criteria for Adverse Events (CTCAE) version 4.0 and coded to preferred terms using Medical Dictionary for Regulatory Activities version 22.1. Treatment-emergent AEs (TEAEs) were defined as those with an onset date or a pre-existing AE worsening following the first dose of study treatment through to 30 days after the last dose of study treatment. Prespecified AEs of special interest (AESI) for olaparib were myelodysplastic syndrome (MDS) or acute myeloid leukemia (AML), a new primary malignancy (other than MDS/AML), and pneumonitis.

### Statistical analyses

The primary and secondary clinical effectiveness outcomes are reported for all patients in the gBRCAm cohort who received at least one dose of olaparib. Safety outcomes are summarized for all patients (gBRCAm and sBRCAm) who received at least one dose of olaparib. The final analysis was planned after reaching at least 130 OS events (approximately 52% data maturity) in the gBRCAm cohort. The sample size estimate for OS was based on recruitment of 250 patients with a germline BRCA mutation; if median OS was 19 months and analyzed after 130 OS events, the 95% confidence interval (CI) for the median would be predicted to extend from 16.0 to 22.6 months (based on the formula of Collett) [33].

The Kaplan–Meier method was used to generate survival curves for all time-to-event endpoints (PFS, OS, DoCR, TFST, TSST, TDT, and PFS2), from which estimates of the median were calculated, together with event rates at 6-monthly intervals and their associated 95% CIs. The associated 95% CI for the median was derived based on the Brookmeyer–Crowley method. A 95% CI for CRR was calculated using the Clopper–Pearson exact method for binomial proportions. The median duration of follow-up was determined in patients who were censored.

Prespecified PFS, OS, and CRR subgroup analyses were performed in the gBRCAm cohort according to HR status (HR-positive or TNBC), previous chemotherapy for mBC (yes or no), previous platinum-based chemotherapy for BC (yes or no; the yes category was further classified into neoadjuvant/adjuvant and metastatic), line of therapy (first-line [i.e. no prior chemotherapy in the first-line advanced/metastatic setting but prior chemotherapy in the neoadjuvant/adjuvant setting] versus second line or later [i.e. prior chemotherapy in the first-line advanced-metastatic setting, with or without prior chemotherapy in neoadjuvant/adjuvant setting]) and prior platinum-based chemotherapy (yes or no), and previous cyclin-dependent kinase 4/6 (CDK4/6) inhibitor treatment for BC (yes or no). Previous chemotherapy for mBC was defined as having received at least one but not more than two lines of chemotherapy in the metastatic setting and did not include chemotherapy given in the neoadjuvant/adjuvant setting; previous endocrine therapy that may have been received by some patients for mBC was not taken into consideration. No formal statistical comparisons were performed among subgroups.

## Results

### Patient disposition

Between January 17, 2018, and March 21, 2019, 563 patients were screened, and 256 patients were enrolled (gBRCAm, *n* = 253; sBRCAm, *n* = 3) (Supplementary Fig. 1). One patient in the gBRCAm cohort did not receive olaparib and was excluded from the full analysis set (*n* = 255). Fewer patients were enrolled into the sBRCAm cohort (*n* = 3) than planned (*n* = 20) owing to the short period of time between the protocol amendment that allowed their inclusion and trial completion. At the data cutoff for this final prespecified analysis, 29 (11.5%) patients were still receiving study treatment. The most common reason for discontinuation of olaparib was disease progression (*n* = 192; 75.3%).

### Baseline characteristics

Baseline characteristics for all patients (gBRCAm and sBRCAm; Table 1) were similar to those for the gBRCAm cohort (Supplementary Table 1). Median age of all patients was 45.0 years (range, 22–75 years) and most patients were White (*n* = 177 [69.4%]) (Table 1). Slightly more patients (*n* = 138 [54.1%]) had a *BRCA1* mutation only compared with the 109 patients (42.7%) with a *BRCA2* mutation only; five patients (2.0%) had both *BRCA1* and *BRCA2* mutations. Most patients had an Eastern Cooperative Oncology Group (ECOG) performance status (PS) score of 0 (*n* = 185 [72.5%]) and were initially diagnosed with stage I─II disease according to the American Joint Committee on Cancer (*n* = 135 [52.9%]). The study population was well balanced with regard to HR status. Eleven patients (4.3%) had central nervous system metastases at baseline. In total, 221 patients (86.7%) had previously received an anthracycline and 226 patients (88.6%) had previously received a taxane in any setting; 192 patients (75.3%) had received both an anthracycline and a taxane. Eighty-one patients (31.8%) received previous platinum-based chemotherapy. In total, 48 patients (59.3%) who had received previous platinum-based chemotherapy had TNBC; none of the patients in the sBRCAm cohort had previously received platinum-based chemotherapy. In this study, 117 patients (45.9%; gBRCAm, *n* = 116; sBRCAm, *n* = 1) had previously received chemotherapy for mBC in the first-line setting and 138 patients (54.1%; gBRCAm, *n* = 136; sBRCAm, *n* = 2) had not received chemotherapy in the mBC setting.

**Table 1:** Baseline characteristics (full analysis set)

Baseline characteristic	All patients^a^ (*N* = 255)
Age, years, median (min–max)	45.0 (22–75)
Female, n (%)	251 (98.4)
Race, n (%)	
White Asian Black or African American American Indian or Alaska Native Missing	177 (69.4) 23 (9.0) 2 (0.8) 1 (0.4) 52 (20.4)
ECOG performance status, n (%)	
0 1 2 Missing	185 (72.5) 65 (25.5) 2 (0.8) 3 (1.2)
AJCC stage at diagnosis, n (%)	
I II III IV Missing	37 (14.5) 98 (38.4) 68 (26.7) 43 (16.9) 9 (3.5)
Time from first diagnosis of BC to study entry, months, median (min–max)	46.3 (4–500)
Time from first diagnosis of mBC to study entry, months, median (min–max)	9.4 (0–279)
BRCA mutation type^b^ n (%)	
* BRCA1* * BRCA2* * BRCA1* and *BRCA2* Missing	138 (54.1) 109 (42.7) 5 (2.0) 3 (1.2)
HR status, n (%)	
HR-positive TNBC	136 (53.3) 119 (46.7)
Menopausal status at baseline, n (%)	
Pre-menopausal Peri-menopausal Post-menopausal Not applicable	69 (27.1) 4 (1.6) 178 (69.8) 4 (1.6)
Prior CT for BC, n (%)^c^	
Neoadjuvant Adjuvant One line for mBC Two lines for mBC Missing	112 (43.9) 155 (60.8) 117 (45.9) 4 (1.6) 1 (0.4)
Previous anthracycline-based CT, n (%)	221 (86.7)
Previous taxane-based CT, n (%)	226 (88.6)
Previous anthracycline- and taxane-based CT, n (%)	192 (75.3)
Previous platinum-based CT, n (%)	81 (31.8)
Neoadjuvant/adjuvant	34 (42.0)
Metastatic	44 (54.3)
Neoadjuvant/adjuvant and metastatic	3 (3.7)
Previous CDK4/6 inhibitor therapy, n (%)^d^	25 (18.4)

*AJCC* American Joint Committee on Cancer; *BC* breast cancer; *CDK4/6* cyclin-dependent kinase 4/6; *CT* chemotherapy; *ECOG* Eastern Cooperative Oncology Group; *gBRCA*, germline BRCA-mutated; *HR* hormone receptor; *mBC* metastatic breast cancer; *sBRCAm* somatic BRCA-mutated; *TNBC* triple-negative breast cancer

^a^255 patients were enrolled between January 2018 and March 2019, including three patients with a somatic BRCA mutation who were permitted to enter the study following a protocol amendment (April 27, 2018)

^b^BRCA mutation type for the sBRCAm cohort: *BRCA1* and *BRCA2*, *n* = 1; missing, *n* = 2

^c^The patient with a missing treatment status was in the gBRCAm cohort

^d^Data reported as a percentage of all patients with HR-positive mBC (*n* = 136); all patients who had previously received CDK4/6 inhibitor therapy had HR-positive mBC

### Clinical effectiveness

**Fig. 1:**
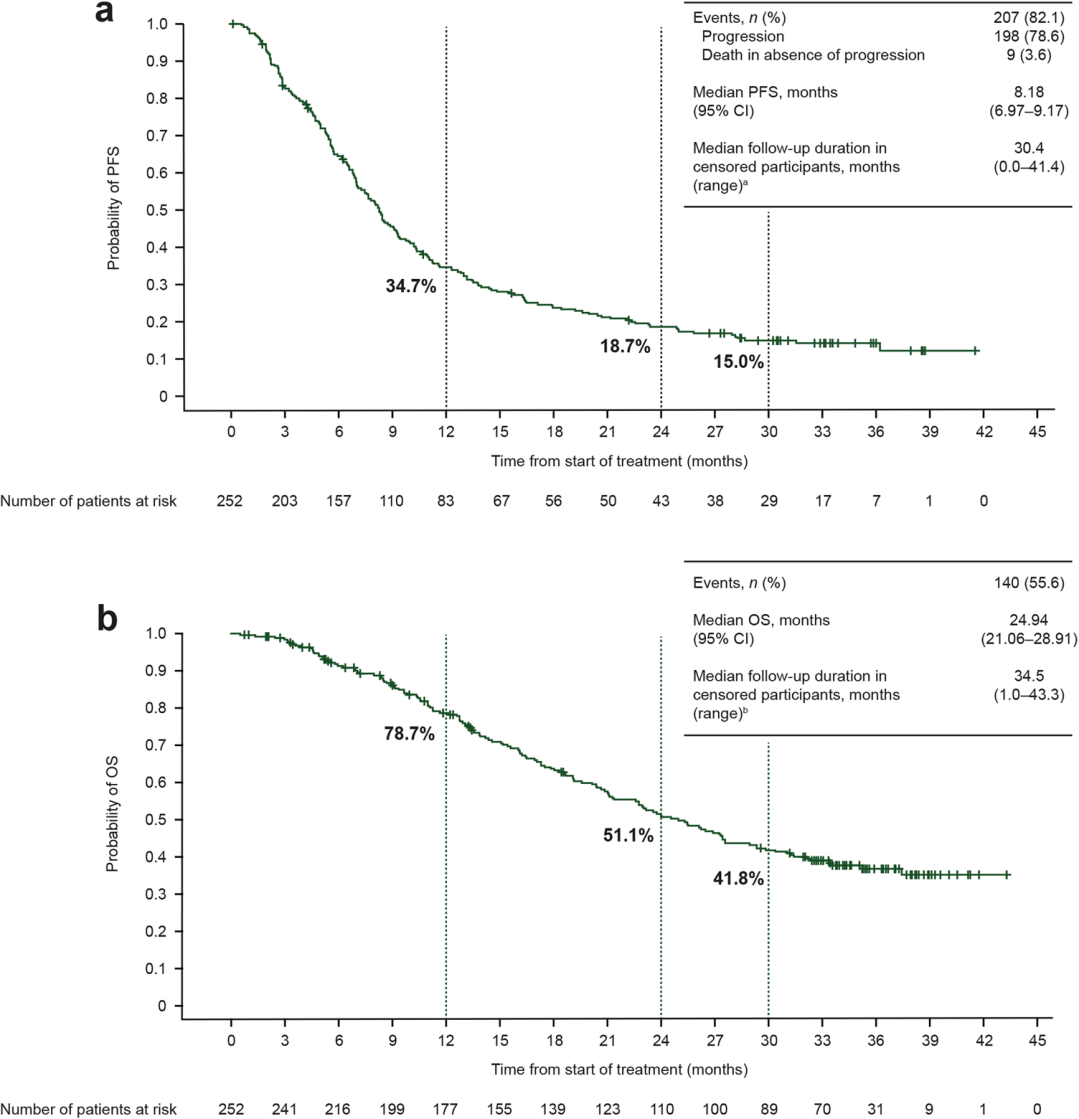
Kaplan–Meier estimates for **a** PFS and **b** OS in the gBRCAm cohort (*N* = 252). Vertical gray dashed lines and corresponding percentages represent the estimated event rates at 12, 24, and 30 months after starting olaparib treatment.  *BRCA* *BRCA1* and/or *BRCA2*; *CI* confidence interval; *gBRCAm* germline BRCA-mutated; *OS* overall survival; *PFS* progression-free survival.  ^a^Reasons for censoring (*n* = 45; 17.9%): progression-free at time of analysis (*n* = 34; 13.5%), lost to follow-up (*n* = 1; 0.4%), withdrawn consent (*n* = 7; 2.8%), terminated study for other reason (*n* = 3; 1.2%).  ^b^Reasons for censoring (*n* = 112; 44.4%): still in survival follow-up (*n* = 79; 31.3%), terminated study before death (*n* = 33; 13.1% [lost to follow-up (*n* = 2; 0.8%), withdrawn consent (*n* = 28; 11.1%), other reasons (*n* = 3; 1.2%)])

At data cutoff, there were 207 PFS events in the gBRCAm cohort (82.1% maturity). The primary endpoint of median PFS was 8.18 months (95% CI 6.97–9.17; Fig. 1a). There were 140 OS events in the gBRCAm cohort (55.6% maturity). Median OS was 24.94 months (95% CI 21.06–28.91 months). (Fig. 1b).

Almost half of the patients in the gBRCAm cohort (*n* = 125 [49.6%]) had a clinical response (Table 2). Median DoCR was 8.0 months (interquartile range, 4.2–18.6 months). Median time to onset of a clinical response was 2.6 months (range, 0.7–30.4 months). Of those with a clinical response, 99 patients (79.2%) subsequently progressed or died. In the gBRCAm cohort, 91 patients (36.1%) had stable disease and 32 patients (12.7%) had progressive disease as their best clinical response. Four patients (1.6%) did not have an evaluable post-baseline assessment.

**Table 2:** Investigator-assessed CRR: overall and by subgroups (gBRCAm cohort)

		N^a^	Patients with clinical response^b^	
			n (%)	95% CI^c^
gBRCAm cohort		252	125 (49.6)	43.3–55.9
Prior CT for mBC^d^				
Yes No		116 136	53 (45.7) 72 (52.9)	36.4–55.2 44.2–61.6
Prior treatment with platinum-containing CT				
Yes Neoadjuvant/adjuvant First-line No		81 34 47 171	34 (42.0) 17 (50.0) 17 (36.2) 91 (53.2)	31.1–53.5 32.4–67.6 22.7–51.5 45.4–60.9
Line of therapy^e^ and prior treatment with platinum-containing CT				
First-line and yes		25	13 (52.0)	31.3–72.2
First-line and no		111	59 (53.2)	43.4–62.7
s-line or later and yes		56	21 (37.5)	24.9–51.5
s-line or later and no		60	32 (53.3)	40.0–66.3
HR status				
HR-positive		134	65 (48.5)	39.8–57.3
TNBC		118	60 (50.8)	41.5–60.2
Prior treatment with a CDK4/6 inhibitor				
Yes No		25 227	15 (60.0) 110 (48.5)	38.7–78.9 41.8–55.2

*BRCA* *BRCA1* and/or *BRCA2*; *CDK4/6* cyclin-dependent kinase 4/6; *CI* confidence interval; *CRR* clinical response rate; *CT* chemotherapy; *gBRCAm* germline BRCA-mutated; *HR* hormone receptor; *mBC* metastatic breast cancer; *TNBC* triple-negative breast cancer

^a^Number of patients who received at least one dose of olaparib

^b^Response did not require confirmation. Responses that occurred after the start of subsequent anti-cancer therapy were not considered

^c^95% CI calculated using the Clopper–Pearson exact method for binomial proportions

^d^Yes: received one or two previous lines of CT for metastatic disease (may have also received CT in the neoadjuvant/adjuvant setting). No: no previous CT for advanced/metastatic disease but received in the neoadjuvant/adjuvant setting

^e^First-line defined as no prior chemotherapy used in the first-line advanced/metastatic setting but received chemotherapy in the neoadjuvant/adjuvant setting. Second-line or later defined as prior chemotherapy used in the first-line advanced-metastatic setting, with or without prior chemotherapy in the neoadjuvant/adjuvant setting

Median TDT and TFST were 7.98 months (95% CI 6.90–8.54) and 9.40 months (95% CI 8.61–10.64), respectively in the gBRCAm cohort (Supplementary Table 2). Median PFS2 and TSST were 14.49 months (95% CI 13.17–17.05) and 14.72 months (95% CI 13.50–17.25), respectively.

**Fig. 2:**
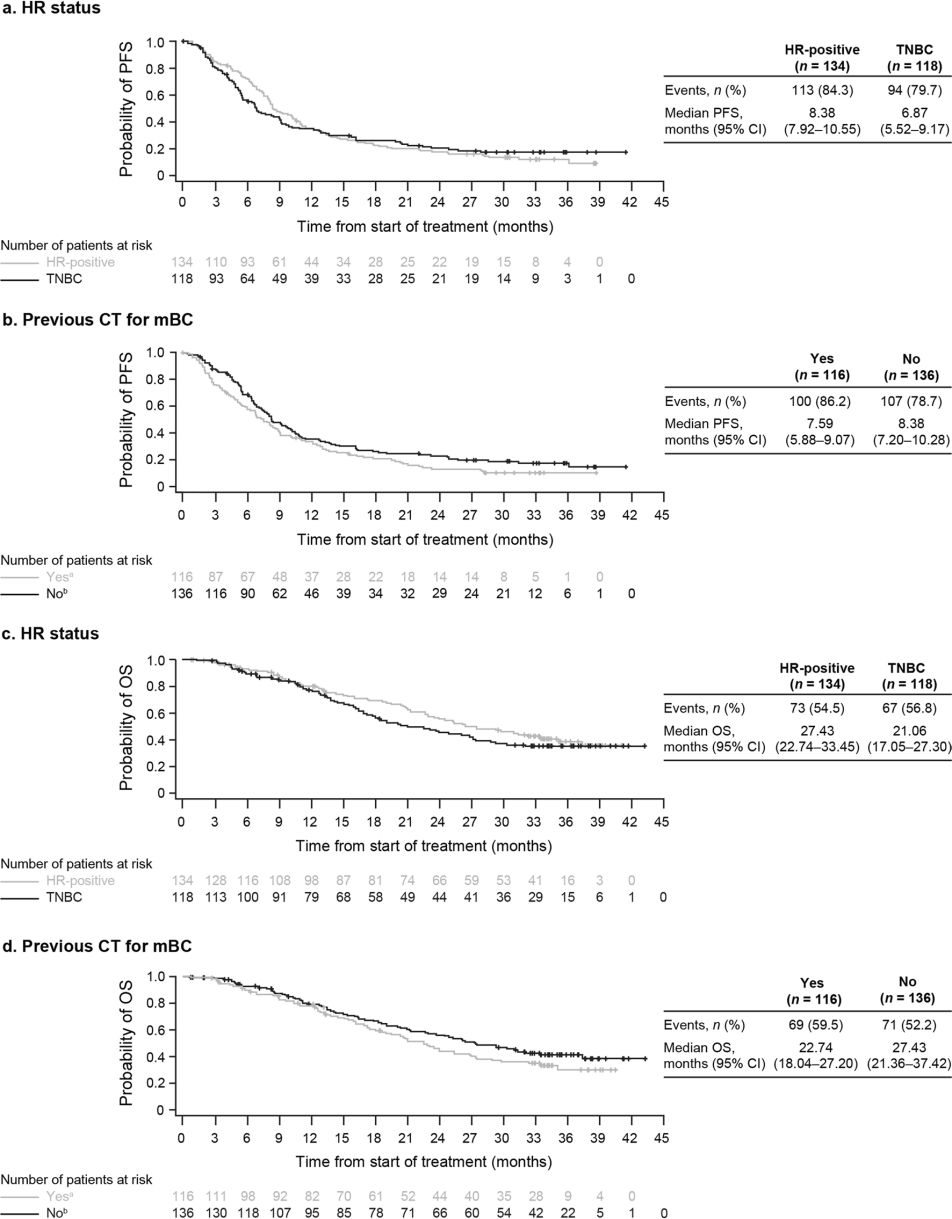
Kaplan–Meier estimates for **a**, **b** PFS and **c**, **d** OS in the gBRCAm cohort by HR status and by previous CT for mBC.  *BRCA* *BRCA1* and/or *BRCA2*; *CI* confidence interval; *CT* chemotherapy; *gBRCAm* germline BRCA-mutated; *HR* hormone receptor; *mBC* metastatic breast cancer; *OS* overall survival; *PFS* progression-free survival; *TNBC* triple-negative breast cancer.  ^a^Yes: received one or two previous lines of CT for metastatic disease (may have also received CT in the neoadjuvant/adjuvant setting).  ^b^No: no previous CT for advanced/metastatic disease but received in the neoadjuvant/adjuvant setting

Olaparib was clinically effective across all predefined subgroups of patients in the gBRCAm cohort evaluated. PFS and OS according to HR status, by previous chemotherapy for mBC, prior exposure to platinum-containing therapy (including by line of therapy), and with prior exposure to CDK4/6 inhibitor (HR-positive mBC) are shown in Fig. 2 and Table 3.

**Table 3:** Progression-free survival and overall survival by subgroups (gBRCAm cohort)

Subgroup	gBRCAm cohort, n	Median PFS, months (95% CI)	Median OS, months (95% CI)
Prior CT for mBC			
Yes^a^	116	7.59 (5.88–9.07)	22.74 (18.04–27.20)
No^b^	136	8.38 (7.20–10.28)	27.43 (21.36–37.42)
Prior treatment with platinum-containing CT			
Yes	81	6.54 (4.93–8.38)	17.28 (13.50–23.98)
Neoadjuvant/adjuvant	34	7.52 (5.39–12.78)	20.83 (12.78–NC)
First-line setting^c^	47	5.19 (3.15–8.18)	17.28 (9.66–23.98)
No	171	9.07 (7.59–10.28)	27.43 (23.52–33.45)
Line of therapy^d^ and prior treatment with platinum-containing CT			
First-line and yes	25	8.34 (6.47–12.91)	16.49 (10.58–NC)
First-line and no	111	8.38 (7.20–10.61)	29.34 (23.75–NC)
Second-line or later and yes	56	5.22 (3.15–8.18)	20.37 (13.34–23.98)
Second-line or later and no	60	9.07 (7.00–12.58)	26.87 (19.09–NC)
HR status			
HR-positive	134	8.38 (7.92–10.55)	27.43 (22.74–33.45)
Triple-negative	118	6.87 (5.52–9.17)	21.06 (17.05–27.30)
HR-positive mBC with prior treatment with a CDK4/6 inhibitor^e^			
Yes	25	9.13 (6.90–14.46)	NC (NC–NC)
No	109	8.38 (7.59–10.28)	NC (NC–NC)

*CI* confidence interval; *CT* chemotherapy; *gBRCAm* germline BRCA-mutated; *mBC* metastatic breast cancer; *NC* not calculated; *OS* overall survival; *PFS* progression-free survival

^a^Yes defined as received one or two previous lines of CT in the mBC setting (may have also received CT in the neoadjuvant/adjuvant setting)

^b^No defined as no previous CT in the advanced/metastatic setting but received in the neoadjuvant/adjuvant setting

^c^*n* = 2 patients received platinum-based CT in the neoadjuvant/adjuvant settings, as well as the first-line setting

^d^First-line defined as no prior CT used in the first-line advanced/metastatic setting but received CT in the neoadjuvant/adjuvant setting. Second-line or later defined as prior CT used in the first-line advanced-metastatic setting, with or without prior CT in the neoadjuvant/adjuvant setting

^e^CDK4/6 inhibitors included palbociclib and ribociclib

CRR in the gBRCAm cohort was similar irrespective of HR status, previous chemotherapy for mBC, previous treatment with platinum-based chemotherapy (including by line of therapy), and previous treatment with a CDK4/6 inhibitor (Table 2).

Clinical effectiveness outcomes for the patients in the exploratory sBRCAm cohort are reported in Supplementary File 2.

### Safety

In total, 165 patients (79.3%) were still receiving study treatment at the time of progression. The median total treatment duration in all patients was similar to the actual median treatment duration (7.98 months; range, 0.2–43.3 months and 7.46 months; range, 0.1–43.3 months, respectively).

**Fig. 3:**
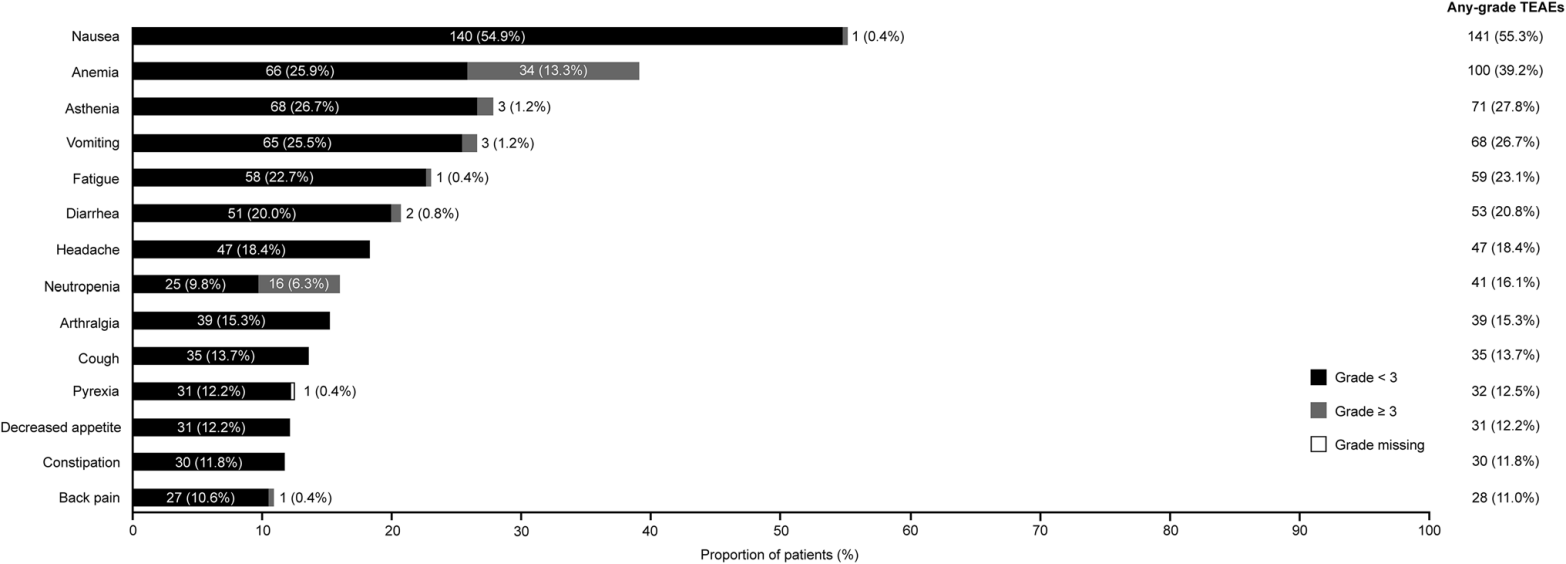
Most frequent TEAEs (occurring in > 10% patients) (full analysis set, *N* = 255). Data are reported as number of patients (%) with: grade < 3 TEAEs (black bars), grade ≥ 3 TEAEs (grey bars) and any-grade TEAEs (right-hand side of graph). TEAEs were graded according to Common Terminology Criteria for Adverse Events version 4.0 and coded to preferred terms using the Medical Dictionary for Regulatory Activities version 24.0. TEAE, treatment-emergent adverse event

Most patients (*n* = 246 [96.5%]) experienced a TEAE. The most frequent TEAEs (occurring in at least 20% of all patients) were nausea, anemia, asthenia, vomiting, fatigue, and diarrhea (Fig. 3). Most TEAEs were CTCAE grade 1 or 2 in severity (*n* = 175 patients [68.6%]); grade 3 or higher TEAEs were reported in 71 patients (27.8%). The most frequent grade 3 or higher TEAEs (reported in at least 2% of patients) were anemia (*n* = 34 [13.3%]) and neutropenia (*n* = 16 [6.3%]). Serious TEAEs were reported in 33 patients (12.9%); the most frequent (occurring in more than one patient) were anemia (*n* = 7 [2.7%]), febrile neutropenia, vomiting, and asthenia (all *n* = 2 [0.8%]). Overall, 182 (71.4% full analysis set) patients required a dose modification of study treatment; dose modification was due to a TEAE in 111 patients (43.5% full analysis set). The most frequent TEAEs (occurring in at least 20 patients) leading to dose modification were anemia (*n* = 55 [21.6%]), neutropenia (*n* = 23 [9.0%]), and vomiting (*n* = 21 [8.2%]). Few patients discontinued study treatment due to a TEAE (*n* = 16 [6.3%]).

Ten patients (3.9%) had AESIs: pneumonitis (*n* = 5) and MDS, bladder cancer in situ (stage 0), neoplasm of the appendix, pancreatic carcinoma, and radiation fibrosis (all *n* = 1). Following database lock, a review of the patient with radiation fibrosis (originally identified as a pneumonitis AESI) determined that the patient did not have an AESI. All pneumonitis TEAEs were grade 2 or less; two cases led to discontinuation of olaparib.

No deaths associated with AEs were reported during the study treatment period or during the 30-day safety follow-up period following the last dose of olaparib. Two deaths (0.8%) associated with AEs were reported after the 30-day safety follow-up period. One death (0.4%) occurred more than 30 days after the last treatment dose and was related to both the disease under investigation and an AE. Supplementary Table 3 summarizes the most frequent treatment-related AEs.

## Discussion

The clinical effectiveness of olaparib in the LUCY trial supports previous findings from the randomized, phase III OlympiAD trial of olaparib versus chemotherapy of physician’s choice in patients with gBRCAm, HER2-negative mBC, underscoring the value of olaparib in this patient population [14, 15].

At the data cutoff for this final prespecified analysis, the median PFS (primary endpoint) in the gBRCAm cohort (8.18 months) was similar to that reported for olaparib in the primary analysis of the OlympiAD trial (7.03 months) [15]. Interestingly, the median OS in the gBRCAm cohort of the LUCY trial (24.94 months) was numerically longer than that reported for olaparib in the final prespecified analysis of OlympiAD (19.25 months) [14]. This difference in survival may reflect the higher proportion of patients in LUCY (~ 54%) compared with OlympiAD (~ 29%) who had not received prior chemotherapy for mBC, which is supported by the longer PFS and OS seen in this subgroup in both studies [14, 15]. However, it should be noted that median OS in patients who had received prior chemotherapy for mBC was also longer in LUCY (22.74 months) than in OlympiAD (18.83 months) [14]. Median total treatment duration and median actual treatment duration were similar in LUCY (8.0 months and 7.5 months, respectively) and OlympiAD (8.2 months and 7.5 months, respectively) [14], suggesting that a difference in exposure to olaparib treatment cannot account for the difference in OS.

The efficacy of olaparib was even more profound in the OlympiA study, where statistically significant improvements in median invasive and distant disease-free survival and OS were observed in patients with HER2-negative, high-risk early BC treated with adjuvant olaparib compared with those who received placebo, further suggesting that earlier treatment with olaparib results in improved efficacy [24, 25]. Targeted treatment would reasonably be expected to offer greatest clinical benefit when there is limited clonal evolution in response to prior treatment [15, 24, 27, 34].

In predefined subgroup analyses, olaparib was clinically effective (as assessed by PFS and OS) in all key subgroups of patients in the gBRCAm cohort, including HR status, previous chemotherapy for mBC, previous platinum-based chemotherapy (including by line of therapy), or previous treatment with CDK4/6 inhibitors. Accordingly, the findings reinforce the clinical efficacy of olaparib in patients with TNBC and HR-positive disease, which is consistent with olaparib targeting the same underlying cause of the disease in these patients. Patients with TNBC and those who received platinum-based chemotherapy had a similar response to olaparib, which was expected owing to the overlap of patient populations in these subgroups. Questions remain regarding the optimal sequencing of treatment with olaparib and CDK4/6 inhibitors. Real-world evidence indicates that patients with gBRCAm who receive CDK4/6 inhibitors as first-line therapy for mBC have poorer treatment outcomes than patients with wild-type BRCA [4, 35]. Accordingly, treatment with olaparib earlier in the disease course may be particularly important in patients with gBRCAm, HER2-negative mBC. The OS, TFST, TSST, and PFS2 findings suggest that olaparib treatment may delay subsequent treatment and progression milestones.

Safety findings were consistent with the well-tolerated and manageable safety profile of olaparib seen in the interim analysis of the LUCY trial and in previous olaparib studies, both in BC and in other tumor types [14, 15, 24, 27]. Although most patients (71.4%) had a dose modification for several reasons, including AEs, the rate of discontinuation from olaparib due to TEAEs was low (6.3%) and similar to that observed in OlympiAD (4.9%) [14]. This suggests that TEAEs were generally effectively managed, allowing sustained treatment with olaparib for as long as patients received a clinical benefit. One patient experienced grade 4 MDS, considered by the investigator to be treatment-related, which led to discontinuation of olaparib treatment. Of note, this patient had previously received both anthracycline- and platinum-based chemotherapy, which are known DNA-damaging agents [36]. It is the only case of MDS that has been reported in the OlympiAD and LUCY trials. To date, no cases of AML have been reported in either trial [14, 15, 27]. In the OlympiA trial in early BC, MDS/AML were reported in two patients in the olaparib arm and three patients in the placebo arm [24]. MDS/AML have also been reported in patients with ovarian and prostate cancer treated with olaparib. These patients had received chemotherapy before exposure to olaparib, which may have included treatments linked to an increased risk of MDS/AML (such as radiotherapy and platinum-based chemotherapy) [36–38].

Differences between LUCY and OlympiAD must be considered when comparing findings from the two trials [27]. The LUCY trial was designed to have less stringent eligibility criteria compared with the OlympiAD trial. For example, in LUCY there were no eligibility criteria related to ECOG-PS, whereas all patients enrolled in OlympiAD had to have an ECOG-PS of 0–1. Interestingly, despite this, most patients (*n* = 250 [98.0%]) enrolled in LUCY had an ECOG-PS of 0–1, perhaps reflecting where physicians see the value of olaparib for patients. Patients enrolled in LUCY were required to have previously received either taxane- or anthracycline-based chemotherapy, whereas patients in OlympiAD must have previously received both taxane- and anthracycline-based chemotherapy. However, 75.3% of patients in LUCY had received both drugs, reflective of standard clinical practice. Therefore, despite the differences in eligibility criteria, the OlympiAD and LUCY trials enrolled similar patient populations that are highly relevant to those encountered in clinical practice. Efficacy measurements differed in the LUCY and OlympiAD studies. Specifically, tumor responses were assessed by the study investigators in the LUCY trial, whereas a more standardized approach was adopted in OlympiAD, where tumor responses were assessed by blinded independent central review [15, 27]. In LUCY, the frequency of patient follow-up for tumor evaluation following a first progression event was not mandated and was instead carried out per local practice and standard of care. This limitation should be taken into consideration when reviewing the intermediate efficacy endpoints within the study.

Other limitations include the limited diversity of the patient population enrolled, suggesting that increased efforts are required to broaden inclusion in clinical trials overall and to better represent the populations at risk. The short period between the protocol amendment permitting the inclusion of patients with an sBRCA mutation (April 27, 2018) and the date of the last patient enrollment (March 21, 2019), as well as the lack of routine screening for sBRCA mutations at the time of the study likely contributed to the low number of patients enrolled into the sBRCAm cohort [35]. As well, the small sample size and missing data limited assessment of the clinical effectiveness of olaparib in patients with an sBRCA mutation. Although no firm conclusions can be drawn from the sBRCAm cohort in this study, a phase II single-arm, proof-of-principle trial has reported encouraging data in mBC [28]. Furthermore, evidence in ovarian and prostate cancer is also supportive of a benefit of PARP inhibition for patients with sBRCA mutations [28–32]. Additional investigations in patients with sBRCA mutations are warranted.

## Conclusions

The findings from the final prespecified analysis of the phase IIIb LUCY trial support the clinical effectiveness and safety of olaparib in patients with gBRCAm, HER2-negative mBC in a real-world setting, and may help to guide clinical practice.

### Supplementary Information

Below is the link to the electronic supplementary material.

Supplementary material 1 (DOCX 278.5 kb)

Supplementary material 2 (DOCX 50.2 kb)

Supplementary material 3 (DOCX 48.1 kb)

Supplementary material 4 (DOCX 55.0 kb)

## Data Availability

Data underlying the findings described in this manuscript may be obtained in accordance with AstraZeneca’s data-sharing policy, described at: https://astrazenecagrouptrials.pharmacm.com/ST/Submission/Disclosure.
