# Supplementary material for: Clinical effectiveness and safety of olaparib in BRCA-mutated, HER2-negative metastatic breast cancer in a real-world setting: final analysis of LUCY
Source: Breast Cancer Res Treat. 2023 Dec 19;204(2):237–48. doi: 10.1007/s10549-023-07165-x (PMC10948524; doi:10.1007/s10549-023-07165-x)
Supplement: Supplementary file 1 — Supplementary material 1 (DOCX 278.5 kb) [file 10549_2023_7165_MOESM1_ESM.docx]

## Supplementary Fig. 1. Patient disposition at the final prespecified analysis data cutoff (September 1, 2021).


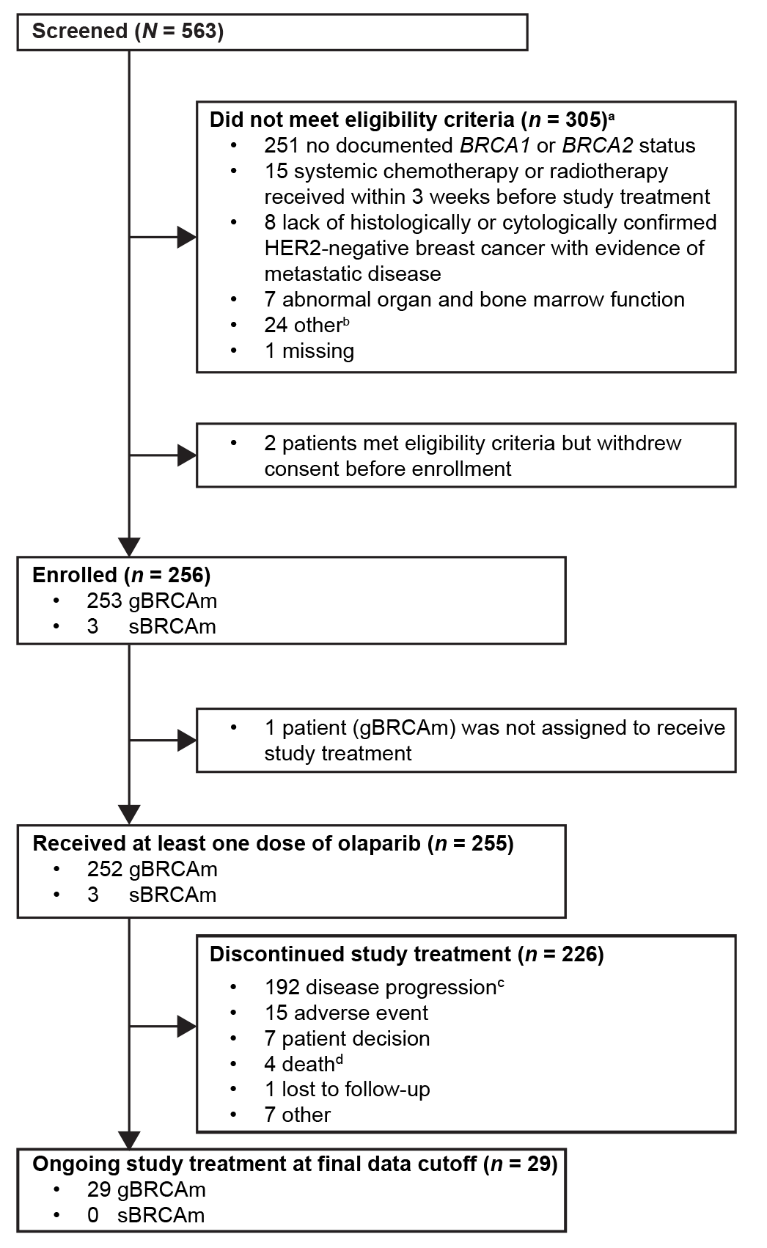


BRCA, *BRCA1* and/or *BRCA2;* gBRCAm, germline BRCA-mutated; HER2, human epidermal growth factor receptor 2; sBRCAm, somatic BRCA mutation.

^a^Eligibility criteria were not mutually exclusive.

^b^Other reasons for exclusion: did not provide informed consent (*n* = 4; 1.3%), received more than one prior line of chemotherapy in the metastatic setting (*n* = 3; 1.0%), received more than two prior cytotoxic chemotherapy regimens in the metastatic setting (*n* = 1; 0.3%), life expectancy < 16 weeks (*n* = 4; 1.3%), not using effective contraception (*n* = 1; 0.3%), unable to comply with the protocol (*n* = 5; 1.6%), other malignancy within the last 5 years (*n* = 2; 0.6%), concomitant use of strong/moderate cytochrome P450, family 3, subfamily A (CYP3A) inhibitors (*n* = 1; 0.3%), symptomatic uncontrolled brain metastases (*n* = 3; 1.0%).

^c^Of 192 patients that discontinued study treatment due to disease progression, n = 3 were from the sBRCAm cohort.

^d^One death was due to COVID-19.
