# Supplementary material for: Clinical effectiveness and safety of olaparib in BRCA-mutated, HER2-negative metastatic breast cancer in a real-world setting: final analysis of LUCY
Source: Breast Cancer Res Treat. 2023 Dec 19;204(2):237–48. doi: 10.1007/s10549-023-07165-x (PMC10948524; doi:10.1007/s10549-023-07165-x)
Supplement: Supplementary file 2 — Supplementary material 2 (DOCX 50.2 kb) [file 10549_2023_7165_MOESM2_ESM.docx]

**Supplementary File 1**

**Clinical effectiveness and safety of olaparib in BRCA-mutated, HER2-negative metastatic breast cancer in a real-world setting: Final analysis of LUCY**

Judith Balmaña, Peter A. Fasching, Fergus J. Couch, Suzette Delaloge,
Intidhar Labidi-Galy, Joyce O’Shaughnessy, Yeon Hee Park, Andrea F. Eisen,
Benoit You, Hughes Bourgeois, Anthony Gonçalves, Zoe Kemp,
Angela Swampillai, Tomasz Jankowski^1^, Joo Hyuk Sohn^1^, Elena Poddubskaya,
Guzel Mukhametshina, Sercan Aksoy, Constanta V. Timcheva, Tjoung-Won Park-Simon, Antonio Antón-Torres, Ellie John, Katherine Baria, Isabel Gibson, Karen A. Gelmon & the LUCY investigators

**Corresponding author:** Dr Karen A. Gelmon; [kgelmon@bccancer.bc.ca](mailto:kgelmon@bccancer.bc.ca)

## Participating countries and LUCY investigators

| **Country** | **LUCY Investigators** |
| --- | --- |
| **Bulgaria** | Tatyana Koynova, Vasil Popov, Constanta Timcheva, Antoaneta Tomova |
| **Canada** | Andrea Eisen, Karen Gelmon, Julie Lemieux |
| **France** | Paule Augereau^*^, Fernando Bazan, Célia Becuwe, Hugues Bourgeois, Camille Chakiba, Mohamad Chehimi, Caroline Cheneau, Florence Dalenc, Eléonore de Guillebon, Thibault De La Motte Rouge,  Jean-Sébastien Frenel, Anthony Gonçalves, Julien Grenier, Anne Claire Hardy-Bessard, Regine Lamy, Christelle Levy, Alain Lortholary, Audrey Mailliez, Jacques Medioni, Anne Patsouris, Dominique Spaeth, Luis Teixeira, Isabelle Tennevet, Laurence Venat-Bouvet, Cristian Villanueva, Benoit You |
| **Germany** | Johannes Ettl, Peter Fasching, Bernd Gerber, Claus Alexander Hanusch, Oliver Hoffmann, Tjoung-Won Park-Simon, Wolfram Malter, Mattea Reinisch, Joke Tio, Pauline Wimberger |
| **Hungary** | Katalin Boer, Magdolna Dank |
| **Italy** | Alberto Ballestrero, Giampaolo Bianchini; Laura Biganzoli,  Roberto Bordonaro, Francesco Cognetti, Enrico Cortesi, Michelino De Laurentiis, Sabino De Placido, Luca Gianni, Valentina Guarneri, Paulo Marchetti, Filippo Montemurro, Anna Maria Mosconi, Giuseppe Naso, Fabio Puglisi, Armando Santoro, Claudio Zamagni |
| **Japan** | Hiroji Iwata, Seung-Jin Kim, Seigo Nakamura |
| **Korea** | Yee Soo Chae, Eun Kyung Cho, Jee Hyun Kim, Seock-Ah Im,  Keun Seok Lee, Yeon Hee Park, Joo Hyuk Sohn |
| **Poland** | Tomasz Byrski, Tomasz Huzarski, Tomasz Jankowski, Bozena Kukielka-Budny, Aleksandra Lacko, Zbigniew Nowecki, Elzbieta Senkus-Konefka, Renata Szoszkiewicz, Rafal Tarnawski |
| **Russia** | Timur Andabekov, Mikhail Dvorkin, Viktoria Dvornichenko, Fedor Moiseenko, Guzel Mukhametshina, Elena Poddubskaya, Ekaterina Popova, Anna Tarasova, Dina Sakaeva, Marina Shomova, Anna Vats |
| **Spain** | Bárbara Adamo, Raquel Andrés Conejero, Antonio Antón Torres, Judith Balmaña Gelpi, Blanca Cantos Sánchez de Ibarguen, Josefina Cruz Jurado, Nieves Díaz Fernández, Alejandro Falcón González, Juan Garcia, Santiago González Santiago, Fernando Henao Carrasco, Isabel Lorenzo Lorenzo, Fernando Moreno Antón, Beatriz Rojas García, Salomón Menjón Beltrán, Marta Santisteban, Agostina Stradella |
| **Taiwan** | Ming-Feng Hou, Chiun-Sheng Huang, Yung-Chang Lin, Ling-Ming Tseng, Hwei-Chung Wang |
| **Turkey** | Sercan Aksoy, Cagatay Arslan, Mehmet Artac, Adnan Aydiner, Umut Disel, Metin Ozkan, Ozgur Ozyilkan, Emel Yaman Sezer, Tarkan Yetisyigit |
| **UK** | Anne Armstrong, Sophie Barrett, Annabel Borley, Zoe Kemp,  Caroline Michie, Mukesh Mukesh, Timothy Perren, Angela Swampillai |
| **USA** | Madhu Chaudhry, Tammy Young |
