# Supplementary material for: Clinical effectiveness and safety of olaparib in BRCA-mutated, HER2-negative metastatic breast cancer in a real-world setting: final analysis of LUCY
Source: Breast Cancer Res Treat. 2023 Dec 19;204(2):237–48. doi: 10.1007/s10549-023-07165-x (PMC10948524; doi:10.1007/s10549-023-07165-x)
Supplement: Supplementary file 3 — Supplementary material 3 (DOCX 48.1 kb) [file 10549_2023_7165_MOESM3_ESM.docx]

**Supplementary File 2**

**Clinical effectiveness and safety of olaparib in BRCA-mutated, HER2-negative metastatic breast cancer in a real-world setting: Final analysis of LUCY**

Judith Balmaña, Peter A. Fasching, Fergus J. Couch, Suzette Delaloge,
Intidhar Labidi-Galy, Joyce O’Shaughnessy, Yeon Hee Park, Andrea F. Eisen,
Benoit You, Hughes Bourgeois, Anthony Gonçalves, Zoe Kemp,
Angela Swampillai, Tomasz Jankowski^1^, Joo Hyuk Sohn^1^, Elena Poddubskaya,
Guzel Mukhametshina, Sercan Aksoy, Constanta V. Timcheva, Tjoung-Won Park-Simon, Antonio Antón-Torres, Ellie John, Katherine Baria, Isabel Gibson, Karen A. Gelmon & the LUCY investigators

**Corresponding author:** Dr Karen A. Gelmon; kgelmon@bccancer.bc.ca

**Supplementary Results**

## Descriptive analyses of patients in the exploratory somatic BRCA-mutated (sBRCAm) cohort (*n* = 3)

Three patients with a somatic BRCA mutation were enrolled in the LUCY trial and received at least one dose of olaparib. At the data cutoff, all three patients (100%) had discontinued study treatment owing to disease progression; two patients (66.7%) had died and one patient (33.3%) was ongoing in survival follow-up. The median age of this cohort was 56.0 years (range, 42–68 years). All patients had an Eastern Cooperative Oncology Group (ECOG)-performance score (PS) score of 1 (restricted activity). Two patients were initially diagnosed with stage IIA disease (according to American Joint Committee on Cancer); one patient was initially diagnosed with stage III disease. One patient had received previous chemotherapy for metastatic breast cancer as first-line therapy and all three patients had received chemotherapy in the neoadjuvant/adjuvant setting. All patients had previously received both anthracycline- and taxane-based chemotherapy. No patient in this cohort had been previously exposed to platinum-based chemotherapy.

All patients with a somatic BRCA mutation had progressive disease at the end of the study. Two patients were censored because the intervals between tumor assessments were too long and were, therefore, not counted as having progression free survival (PFS) events. One patient (33.3%) had a PFS event (progression or death). All three patients (100%) had a time to first subsequent treatment or death event and a time to discontinuation of study treatment event. Two patients (66.7%) had a time to second subsequent treatment or death event and a time to second progression or death event. No adverse events of special interest were reported for patients in this cohort.

## Adverse event management

Treatment-emergent adverse events (TEAEs) were managed by interrupting or reducing the dose of study treatment. In the full analysis set (germline BRCA-mutated and sBRCAm), 99 patients (38.8%) experienced a TEAE that led to dose interruption. The most frequent TEAEs resulting in dose interruption were anemia (*n* = 51 [20.0%]), neutropenia (*n* = 21 [8.2%]), vomiting (*n* = 12 [4.7%]), and nausea (*n* = 10 [3.9%]). A total of 111 patients (43.5%) experienced a TEAE that led to dose modification. The most frequent TEAEs resulting in dose modification were anemia (*n* = 55 [21.6%]), neutropenia (*n* = 23 [9.0%]), vomiting (*n* = 21 [8.2%]), and nausea (*n* = 15 [5.9%]). Overall, a total of 43 patients (16.9%) received blood transfusions for anemia (grade 2 or higher). Antiemetic therapy was received by 52 patients (20.4%) for nausea and 25 patients (9.8%) for vomiting.
