# Supplementary material for: Clinical effectiveness and safety of olaparib in BRCA-mutated, HER2-negative metastatic breast cancer in a real-world setting: final analysis of LUCY
Source: Breast Cancer Res Treat. 2023 Dec 19;204(2):237–48. doi: 10.1007/s10549-023-07165-x (PMC10948524; doi:10.1007/s10549-023-07165-x)
Supplement: Supplementary file 4 — Supplementary material 4 (DOCX 55.0 kb) [file 10549_2023_7165_MOESM4_ESM.docx]

## Supplementary Table 1. Baseline characteristics (gBRCAm cohort)

| Baseline characteristic | gBRCAm cohort (*N* = 252)^a^ |
| --- | --- |
| Age, years, median (min–max) | 45.0 (22–75) |
| Female, n (%) | 248 (98.4) |
| Race, n (%)  White  Asian  Black or African American  American Indian or Alaska Native  Missing | 177 (70.2)  22 (8.7)  2 (0.8)  1 (0.4)  50 (19.8) |
| ECOG performance status, n (%) |  |
| 0  1  2  Missing | 185 (73.4)  62 (24.6)  2 (0.8)  3 (1.2) |
| AJCC stage at diagnosis, n (%) |  |
| I  II  III  IV  Missing | 37 (14.7)  96 (38.1)  67 (26.6)  43 (17.1)  9 (3.6) |
| Time from first diagnosis of BC to study entry, months, median (min–max) | 45.9 (4–500) |
| Time from first diagnosis of mBC to study entry, months, median (min–max) | 9.3 (0–279) |
| BRCA mutation type, n (%)  *BRCA1*  *BRCA2*  *BRCA1* and *BRCA2*  Missing | 138 (54.8)  109 (43.3)  4 (1.6)  1 (0.4) |
| HR status, n (%)  HR-positive  TNBC | 134 (53.2)  118 (46.8) |
| Menopausal status at baseline, n (%)  Pre-menopausal  Peri-menopausal  Post-menopausal  Not applicable | 68 (27.0)  4 (1.6)  176 (69.8)  4 (1.6) |
| Previous CT for BC, n (%)  Neoadjuvant  Adjuvant  One line for mBC  Two lines for mBC  Missing | 111 (44.0)  153 (60.7)  116 (46.0)  4 (1.6)  1 (0.4) |
| Previous anthracycline-based CT, n (%) | 218 (86.5) |
| Previous taxane-based CT, n (%) | 223 (88.5) |
| Previous platinum-based CT, n (%) | 81 (32.1) |
| Neoadjuvant/adjuvant | 34 (42.0) |
| Metastatic | 44 (54.3) |
| Neoadjuvant/adjuvant and metastatic | 3 (3.7) |
| Previous CDK4/6 inhibitor therapy, n (%)^b^ | 25 (18.7) |

AJCC, American Joint Committee on Cancer; BC, breast cancer; CDK4/6, cyclin-dependent kinase 4/6; CT, chemotherapy; ECOG, Eastern Cooperative Oncology Group; gBRCAm, germline BRCA-mutated; HR, hormone receptor; mBC, metastatic breast cancer; TNBC, triple-negative breast cancer.

^a^Number of patients who received at least one dose of study treatment in the gBRCAm cohort.

^b^Data reported as a percentage of all the patients in the gBRCAm cohort with HR-positive mBC (*n* = 134); all patients who had previously received CDK4/6 inhibitor therapy had HR-positive mBC.

## Supplementary Table 2. Summary of TFST, TSST, TDT, and PFS2 in the gBRCAm cohort

| Outcome | gBRCAm cohort (*N* = 252) |
| --- | --- |
| TFST |  |
| Events, n (%) | 193 (76.6) |
| Censored patients, n (%) | 59 (23.4) |
| Median TFST, months (95% CI) | 9.40 (8.61–10.64) |
| Event-free at 12 months, % (95% CI) | 37.9 (31.7–44.1) |
| Event-free at 24 months, % (95% CI) | 23.6 (18.3–29.3) |
| Event-free at 30 months, % (95% CI) | 19.4 (14.5–24.8) |
| Median duration of follow-up, months (range)^a^ | 32.6 (0.8–43.3) |
| TSST |  |
| Events, n (%) | 171 (67.9) |
| Censored patients, n (%) | 81 (32.1) |
| Median TSST, months (95% CI) | 14.72 (13.50–17.25) |
| Event-free at 12 months, % (95% CI) | 63.1 (56.5–68.9) |
| Event-free at 24 months, % (95% CI) | 32.8 (26.7–39.1) |
| Event-free at 30 months, % (95% CI) | 26.1 (20.5–32.1) |
| Median duration of follow-up, months (range)^a^ | 32.5 (0.8–43.3) |
| TDT |  |
| Events, n (%) | 223 (88.5) |
| Censored patients, n (%) | 29 (11.5) |
| Median TDT, months (95% CI) | 7.98 (6.90–8.54) |
| Event-free at 12 months, % (95% CI) | 32.9 (27.2–38.8) |
| Event-free at 24 months, % (95% CI) | 17.9 (13.4–22.8) |
| Event-free at 30 months, % (95% CI) | 14.7 (10.6–19.3) |
| Median duration of follow-up, months (range)^a^ | 36.4 (31.1–43.3) |
| PFS2 |  |
| Events, n (%) | 168 (66.7) |
| Censored patients, n (%) | 84 (33.3) |
| Median PFS2, months (95% CI) | 14.49 (13.17–17.05) |
| Event-free at 12 months, % (95% CI) | 64.5 (57.9–70.3) |
| Event-free at 24 months, % (95% CI) | 32.8 (26.6–39.2) |
| Event-free at 30 months, % (95% CI) | 26.3 (20.5–32.5) |
| Median duration of follow-up, months (range)^a^ | 26.2 (0.0–41.4) |

The Kaplan–Meier method was used to calculate estimates of the median, together with event rates at six monthly intervals and their associated 95% CI. The 95% CI for the median was calculated using the Brookmeyer–Crowley method.

BRCA, *BRCA1* and/or *BRCA2*; CI, confidence interval; gBRCAm, germline BRCA-mutated; PFS2, time to second progression or death; TDT, time to study treatment discontinuation or death; TFST, time to first subsequent treatment or death; TSST, time to second subsequent treatment or death.

^a^Median duration of follow-up in censored patients.

## Supplementary Table 3. TRAEs occurring in at least 10% of all patients by maximum reported CTCAE grade (full analysis set, *N* = 255)

|  | **All grades** | **Grade 3 or higher** |
| --- | --- | --- |
| Any TRAE | 217 (85.1) | 45 (17.6) |
| Nausea | 122 (47.8) | 0 |
| Anemia | 88 (34.5) | 30 (11.8) |
| Asthenia | 53 (20.8) | 1 (0.4) |
| Fatigue | 46 (18.0) | 0 |
| Vomiting | 45 (17.6) | 0 |
| Neutropenia | 39 (15.3) | 14 (5.5) |
| Diarrhea | 28 (11.0) | 0 |

Data are number of patients (%). Adverse events were considered causally related to study treatment as assessed by the investigator. TRAEs were graded according to CTCAE version 4.0 and coded to preferred terms using the Medical Dictionary for Regulatory Activities version 24.0.

CTCAE, Common Terminology Criteria for Adverse Events; TRAE, treatment-related adverse event.
